# PriorNav: Prior Knowledge Enhanced Zero-Shot Goal Navigation via Multi-Step Iterative Reasoning

**DOI:** 10.3390/s26103057

**Published:** 2026-05-12

**Authors:** Wen Liu, Xuanshun Zhuang, Lei Ma, Zhongliang Deng

**Affiliations:** School of Electronic Engineering, Beijing University of Posts and Telecommunications, Beijing 100876, China; zxs2024@bupt.edu.cn (X.Z.); malei@bupt.edu.cn (L.M.); a20251121@bupt.edu.cn (Z.D.)

**Keywords:** zero-shot goal navigation, prior knowledge, scene graph, iterative reasoning, embodied navigation

## Abstract

Zero-shot goal navigation requires an agent to locate targets in unseen environments based on object categories, reference images, or text descriptions, placing high demands on scene understanding and reasoning. Existing methods mainly rely on online observations, modality similarity, or heuristic graph matching, and therefore still struggle with complex target search due to limited use of external knowledge and weak multi-step reasoning. We propose PriorNav, a prior-knowledge-enhanced framework for zero-shot goal navigation. PriorNav learns a unified retrievable knowledge space from semantic, instance, and relational knowledge, maintains a knowledge-enhanced scene graph by fusing retrieved priors with online observations, and performs progressive decision-making through multi-step iterative reasoning across exploration, verification, and approach stages. Experiments on Object-Goal, Image-Instance Goal, and Text-Goal navigation show that PriorNav improves the success rate over the baseline by 3.5%, 13.3%, and 3.5%, respectively, while also outperforming the strongest training-free baselines on all three tasks. Ablation studies further verify the effectiveness of multi-level prior knowledge, scene-graph enhancement, and iterative reasoning. These results show that combining prior knowledge with explicit reasoning is a promising direction for improving zero-shot goal navigation.

## 1. Introduction

Goal-directed navigation in unseen environments is a fundamental capability for embodied agents operating in realistic indoor scenes. In zero-shot goal navigation, an agent must search for and reach a target specified by an object category, a reference image, or a textual description, without task-specific adaptation to the test environment. Compared with classical instruction-following vision-language navigation, this setting places greater demands on target grounding, semantic understanding, cross-modal alignment, and efficient exploration under partial observability. These challenges make zero-shot goal navigation an important testbed for studying how embodied systems can generalize beyond memorized trajectories and exploit high-level knowledge during decision-making [1]. With the availability of large-scale 3D simulation platforms and scene datasets such as Matterport3D [2] and Habitat [3], zero-shot embodied navigation has become an increasingly active research topic.

Recent zero-shot navigation methods have made encouraging progress by leveraging online visual observations, cross-modal similarity, frontier-based exploration, scene graphs, and foundation-model reasoning. However, several important limitations remain. First, many approaches still rely heavily on online observations and local matching, which often leads to inefficient trial-and-error exploration in complex scenes. Second, although some methods introduce graph structures or prompt-based reasoning, decision-making is still frequently dominated by single-step scoring rather than progressive inference over evolving environmental context. Third, scene representations built purely from online perception are vulnerable to occlusion, missed detections, and incomplete relational cues, which can weaken target localization and reduce robustness in unseen environments. As a result, current methods still struggle to effectively integrate prior knowledge, structured scene understanding, and multi-stage reasoning into a unified navigation framework.

To address these issues, we propose PriorNav, a prior-knowledge-enhanced framework for zero-shot goal navigation. The key idea is to combine three complementary capabilities within a closed-loop decision process. First, we construct a retrievable prior knowledge space that unifies semantic knowledge, instance-level knowledge, and relational knowledge, enabling the agent to access target-relevant priors beyond immediate observations. Specifically, we construct a Prior Knowledge Learning Module (PKLM), which extracts semantic knowledge, instance knowledge, and relational knowledge from multiple sources, including WordNet [4], ConceptNet [5], Visual Genome [6], and statistical patterns from training environments. These heterogeneous knowledge entries are encoded into a unified embedding space through contrastive learning, forming a searchable multi-level knowledge base. Second, we maintain a knowledge-enhanced scene graph that fuses retrieved priors with online perceptual evidence, providing a structured and dynamically updated representation of objects, attributes, and relations in the environment. Third, we introduce a multi-step iterative reasoning navigator that progressively refines action decisions across exploration, verification, and approach stages. In this way, PriorNav moves beyond purely perception-driven target search and instead performs navigation by jointly exploiting prior knowledge, structured scene representation, and explicit stage-aware reasoning.

The main contributions of this work are as follows:

- We propose a multi-level prior knowledge learning and retrieval mechanism that unifies semantic, instance-level, and relational knowledge for zero-shot goal navigation, allowing the agent to exploit external priors in a dynamic and target-aware manner.
- We develop a knowledge-enhanced scene graph construction framework that integrates retrieved knowledge with online observations, improving scene completeness, relation modeling, and goal-relevant representation alignment.
- We introduce a stage-adaptive multi-step iterative reasoning strategy that supports progressive decision-making across exploration, verification, and approach stages, leading to more reliable target search in unseen environments.
- We conduct extensive experiments on Object-Goal, Image-Instance Goal, and Text-Goal navigation, showing that PriorNav consistently outperforms representative baselines and that its gains arise from the synergy of prior knowledge, structured scene modeling, and iterative reasoning.

## 2. Related Work

### 2.1. Vision-Language Navigation

Vision-Language Navigation (VLN) aims to enable an embodied agent to navigate in real-world environments by following natural language instructions, and has become an important research direction in embodied intelligence. The Room-to-Room (R2R) dataset [1] was the first to systematically establish a standard research paradigm for this task, and, built upon Matterport3D scenes [2], it promoted the study of language-guided navigation in realistic indoor environments [1,2]. Early approaches mainly adopted sequence-to-sequence frameworks, in which visual observations and language instructions were encoded to directly predict actions. Among them, Speaker-Follower significantly improved model generalization by introducing an instruction generation module for data augmentation [7]. Subsequently, research attention gradually shifted toward pretraining and cross-modal representation learning. PREVALENT was the first to systematically introduce the pretraining–finetuning paradigm into VLN, while VLN-BERT further demonstrated the effectiveness of temporally aware vision-language pretrained models for navigation decision-making [8,9]. Building on this line of work, HAMT improved long-horizon trajectory understanding through hierarchical history modeling, and DUET further combined a global topological map with local observations to achieve dual-scale planning and fine-grained language alignment [10,11]. More recent studies [12,13,14] have further explored memory augmentation, explicit reasoning, and large-model-based planning in embodied navigation, indicating a gradual shift from pure instruction following toward more general decision-making under partial observability. In recent years, surveys in the era of foundation models have also pointed out that VLN has gradually evolved from traditional supervised learning into a unified research paradigm that integrates large models, environmental memory, and structured reasoning. However, existing methods still largely rely on in-distribution experience and online observations, and when faced with complex semantic target search in unseen environments, they remain limited by insufficient environment understanding and inadequate reasoning capability [15]. Therefore, although VLN provides important foundations for cross-modal grounding and sequential decision-making, it does not directly resolve the challenges of zero-shot goal search across object, image, and text goal modalities.

### 2.2. Structured Scene Representation and Prior Knowledge for Navigation

To enhance a navigation system’s understanding of environmental structure, researchers have increasingly introduced structured scene representations, particularly scene graphs, open-vocabulary 3D scene representations, and graph-driven task planning mechanisms. Early work on 3D Scene Graphs demonstrated that graph structures can provide a unified representation of objects, spatial relations, and camera viewpoints within a scene, offering a more compact and interpretable basis for high-level semantic understanding [16]. In the VLN domain, Language and Visual Entity Relationship Graph further jointly modeled linguistic entity relations and visual relations, highlighting the importance of relational information for understanding complex instructions and perceiving environments [17]. Subsequently, OVSG and ConceptGraphs incorporated open-vocabulary capabilities into 3D scene graphs, enabling systems to query target entities or relations using free-form text, thereby improving their applicability to unseen categories and open-ended semantic settings [18,19]. Meanwhile, SayPlan demonstrated the feasibility of grounding high-level plans generated by large language models into real environments through 3D scene graphs, showing the significant value of structured representations for long-horizon task planning [20]. These studies suggest that structured scene representations are valuable not only for semantic abstraction, but also for maintaining relational context, improving interpretability, and supporting downstream reasoning during navigation.

In the direction of prior knowledge, some methods have begun to explicitly incorporate commonsense knowledge and scene statistics. For example, Knowledge-driven Environmental Dreamer improved imagination and reasoning during navigation through knowledge-driven environment modeling, while ESC combined pretrained vision-language models with commonsense language models and transformed room–object relations as well as object co-occurrence knowledge into exploration strategies through soft constraints [21,22]. Related efforts [23,24] have also shown that commonsense priors, object co-occurrence patterns, and room-level regularities can provide useful guidance for narrowing the search space and improving exploration efficiency in unseen environments. Overall, existing studies have shown that both structured scene representations and prior knowledge can effectively reduce the search space and improve navigation interpretability and exploration efficiency. However, most current methods focus only on online graph construction or the use of prior knowledge at a single level, and still lack a unified framework for modeling, dynamically integrating, and continuously reasoning over semantic knowledge, instance knowledge, and relational knowledge. In particular, existing approaches rarely combine multi-level prior retrieval, dynamic scene-graph enhancement, and stage-aware reasoning within a single closed-loop navigation process, which is a key distinction of our work.

### 2.3. Zero-Shot Navigation with Foundation Models

With the development of vision-language models and large language models, zero-shot object navigation has gradually become a key direction in embodied intelligence. ZSON demonstrated the possibility of open-world target search without task-specific ObjectNav rewards or demonstrations by transferring multimodal goal embeddings learned from image-Instance goal navigation to ObjectNav [25]. Subsequently, CoWs [26] on Pasture systematically discussed the benchmarks and challenges of language-driven zero-shot object navigation, showing that CLIP-like models have strong potential for locating rare objects and open-vocabulary targets, but still exhibit clear limitations in reasoning over complex descriptions [26]. How to Not Train Your Dragon [27] further showed that a training-free paradigm combining geometric frontier exploration, semantic point clouds, and spatial scene graphs can achieve strong object navigation performance without reinforcement learning training [27]. These works established important zero-shot and training-free baselines, but they mainly rely on similarity matching, frontier exploration, or heuristic spatial reasoning, rather than explicitly modeling retrievable prior knowledge.

Among foundation model-driven methods, OpenFMNav [28] leveraged large language models and large vision-language models to enable open-set language-guided target navigation, while VLFM [29] constructed a frontier value map based on vision-language similarity, allowing exploration behavior to be explicitly guided by semantic constraints [28,29]. Works based on graph representations and large-model reasoning have further advanced this direction. SG-Nav [30] improved the interpretability and accuracy of zero-shot ObjectNav through online 3D scene graphs and hierarchical chain-of-thought reasoning, while NavGPT and MapGPT enhanced the long-horizon decision-making ability of foundation models in navigation through explicit reasoning chains and map-guided prompting, respectively [12,13,30]. The recent UniGoal [31] pushed this line of research further by using a unified graph representation to simultaneously handle object goals, instance image goals, and text goals, indicating that graph matching and multi-stage exploration are important paths toward general zero-shot target navigation [31]. Nevertheless, existing methods in this line mainly emphasize modality similarity, prompt engineering, graph matching, or reasoning over online observations, and only limited attention has been paid to systematically organizing external prior knowledge into a retrievable representation that can be dynamically fused with scene understanding.

However, most existing methods still rely primarily on modality similarity, prompt engineering, or heuristic graph matching for decision-making, and remain insufficient in the systematic modeling of external prior knowledge and the utilization of multi-step iterative reasoning. This gap constitutes the main motivation of our work. Compared with these methods, PriorNav explicitly unifies semantic, instance-level, and relational priors, injects them into a knowledge-enhanced scene graph, and further performs stage-adaptive iterative reasoning, thereby providing a more integrated solution for zero-shot goal navigation across multiple goal modalities.

## 3. Methods

**Figure 1:**
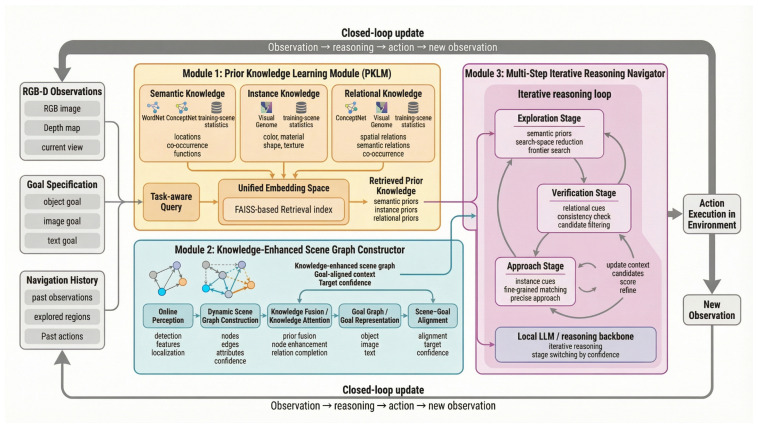
Overview of the proposed PriorNav framework. The system takes RGB-D observations, goal inputs (object, image, or text), and navigation history as input, and performs zero-shot goal navigation through three tightly coupled modules. Module 1 is the Prior Knowledge Learning Module (PKLM), which organizes semantic, instance-level, and relational knowledge from multiple sources, encodes them into a unified embedding space, and supports efficient retrieval via a FAISS-based index. Module 2 is the Knowledge-Enhanced Scene Graph Constructor, which builds an online scene graph from object detection and feature extraction, fuses retrieved prior knowledge through a knowledge attention mechanism, and aligns the scene representation with the goal graph. Module 3 is the Multi-Step Iterative Reasoning Navigator, which uses structured context and retrieved knowledge to perform coarse-to-fine reasoning with a locally deployed LLaMA-2-7B, and adaptively switches among exploration, verification, and approach stages according to target confidence. By integrating knowledge retrieval, structured scene modeling, and stage-adaptive reasoning into a closed-loop pipeline, PriorNav enables more effective target search in unseen environments.

We propose PriorNav, a prior-knowledge-enhanced framework for zero-shot goal navigation. As shown in Figure 1, the framework includes three components: prior knowledge learning and retrieval, knowledge-enhanced scene graph construction, and multi-step iterative reasoning for action selection. Rather than making navigation decisions solely from the current observation, PriorNav organizes external prior knowledge, online scene understanding, and stage-adaptive reasoning into a unified closed-loop decision process. Specifically, at each time step, the agent first takes the current RGB-D observation, goal specification, and navigation history as input, and constructs a task-aware query to retrieve relevant semantic, instance-level, and relational knowledge from the external knowledge base. This knowledge base is built from multiple heterogeneous sources and indexed through a FAISS-based retrieval structure, enabling efficient access to target-relevant priors during navigation. The retrieved knowledge is then fused with online perceptual results to construct a knowledge-enhanced scene graph, which explicitly models objects, attributes, and relations in the current environment and further aligns them with the goal representation. Based on this structured scene representation, the navigator performs coarse-to-fine iterative reasoning to progressively refine action decisions across exploration, verification, and approach stages. As the agent continuously interacts with the environment, the retrieved knowledge, scene graph representation, and reasoning context are all dynamically updated, thereby forming a closed-loop pipeline from observation to action. By coupling these components in a closed loop, PriorNav enables more effective target search in unseen environments.

### 3.1. Problem Formulation

Given a sequence of observations in an unseen environment, denoted as $O=\{o_{1},o_{2},\ldots ,o_{T}\}$, where each observation $o_{t}$ consists of an RGB-D image, pose information, and a locally navigable region, together with a target description *g*, which may take the form of an object category, a reference image, or a textual description, the agent is required to navigate toward the target and reach its location within a limited number of steps. Unlike classical instruction-following vision-language navigation, the task considered in this paper is zero-shot goal navigation, where the target is specified by object, image, or text goals rather than a long navigation instruction. This setting is more challenging because the agent must perform target search and decision-making under partial observability, while also generalizing to previously unseen environments without task-specific adaptation. At each time step, the agent selects an action from the discrete action set A={Move_Forward,Turn_Left,Turn_Right,Stop}. Although the environment interaction is performed through discrete actions, the policy may first evaluate candidate navigable directions at a higher decision level and then map the selected direction to one of the discrete actions. This formulation allows the navigation policy to reason over structured scene context and candidate movement directions before committing to a low-level control action.

First, the test environments are unseen during training. Second, navigation decisions should not rely solely on the current observation, but should also make full use of structured prior knowledge and historical reasoning information accumulated during navigation. Therefore, besides the current observation and target specification, the policy should also leverage an evolving environmental memory and an external prior knowledge base, so that the agent can move beyond purely reactive behavior and make more informed decisions under uncertainty. Therefore, our goal is to learn a policy function

(1) $$a_{t}=\pi \left(o_{t},g,M_{t},K\right)$$

where $M_{t}$ denotes the environmental memory or scene representation at time step *t*, and K denotes the external prior knowledge base. In our framework, $M_{t}$ is instantiated as a dynamically maintained knowledge-enhanced scene graph together with the associated goal-aligned reasoning context, while K contains retrievable semantic, instance-level, and relational priors extracted from external knowledge sources and training-scene statistics. This policy is expected to enable efficient, robust, and interpretable target search in unseen environments.

### 3.2. Prior Knowledge Learning Module

(1) Multi-level Prior Knowledge: To endow the agent with human-like environmental commonsense and spatial reasoning ability, we divide navigation-related prior knowledge into three levels: {semantic knowledge, instance knowledge, and relational knowledge}. The motivation for this design is that zero-shot goal navigation in unseen environments requires complementary guidance at different abstraction levels, rather than relying on a single type of prior. Semantic knowledge provides category-level regularities about where a target is likely to appear and what contextual entities are commonly associated with it, which is particularly useful for reducing the search space during early exploration. Instance knowledge captures appearance-level cues such as color, material, shape, and local texture, which are important for fine-grained matching when the agent needs to distinguish a specific object or verify visually similar candidates. Relational knowledge characterizes stable spatial and semantic dependencies among objects, and therefore helps the agent reason over contextual constraints, object co-occurrence, and scene composition. In this sense, semantic knowledge answers “where to search”, instance knowledge supports “what to match”, and relational knowledge helps determine “how to verify” in complex environments. Semantic knowledge describes the typical attributes, common functions, and frequent occurrence regions of an object category; for example, “a bed is usually located in a bedroom.” Instance knowledge describes the appearance attributes and local visual features of a target instance, such as color, material, shape, and texture. Relational knowledge is used to characterize stable spatial or semantic relationships between objects, such as “chair near table” or “lamp on desk”. Formally, for an object category *c*, the semantic knowledge can be represented as $K^{\mathrm{sem}}\left(c\right)=\left\{L\left(c\right),C\left(c\right),F\left(c\right)\right\}$, where L(c) denotes the set of typical locations, C(c) denotes the set of co-occurring objects, and F(c) denotes the functional attribute descriptions. The relational knowledge can be represented as $K^{\mathrm{rel}}\left(o_{i},o_{j}\right)=\left\{r_{ij}^{\mathrm{spa}},\;r_{ij}^{\mathrm{sem}}\right\}$, where $r_{ij}^{\mathrm{spa}}$ and $r_{ij}^{\mathrm{sem}}$ denote the spatial relation and semantic relation, respectively. By organizing prior knowledge in this hierarchical manner, the knowledge base can simultaneously support category-level target localization, instance-level target discrimination, and relation-level context completion. This is especially valuable in unseen environments, where the agent cannot depend on memorized scene layouts and must instead exploit transferable regularities across scenes. With this hierarchical definition, the knowledge base can simultaneously support category-level inference, instance-level matching, and relation-level completion.

(2) Knowledge Extraction and Embedding: We extract multi-source heterogeneous knowledge from external knowledge bases and training-scene statistics, encoding them into a unified embedding space via contrastive learning [32]. We adopt a unified embedding space instead of storing different knowledge types in separate forms because the navigation process requires the agent to access semantic descriptions, visual attributes, and relational cues in a comparable representation. This shared embedding space makes it possible to retrieve heterogeneous priors in a target-aware and scene-aware manner, while also facilitating their subsequent fusion with online observations. Moreover, such a design improves zero-shot generalization, since the agent can retrieve transferable high-level regularities from external knowledge sources even when the current environment has never been observed during training. Detailed knowledge extraction pipeline, retrieval implementation, and inference configuration are provided in Appendix A.4.

(3) Knowledge Retrieval: During navigation, the agent needs to dynamically retrieve relevant knowledge according to the current goal and environmental state. To this end, we construct a query representation $q_{t}$ based on the current observation and perform nearest-neighbor search in the knowledge base: $K_{t}=\mathrm{TopK}\left(\mathrm{sim}(q_{t},k_{i})\right)$, where sim(·,·) denotes the similarity function, and $K_{t}$ denotes the set of relevant knowledge retrieved at time step *t*. We use a retrievable knowledge space rather than static knowledge injection because only a subset of prior knowledge is useful at a given time step. Static injection would introduce large amounts of irrelevant information and reduce decision focus, whereas dynamic retrieval enables the agent to select priors conditioned on the current goal, perceptual evidence, and navigation stage. As the agent moves through the environment, both the query representation and the retrieved knowledge are continuously updated, allowing knowledge utilization to remain adaptive throughout the navigation process. This mechanism is particularly important for zero-shot navigation, because it enables knowledge transfer across scenes without assuming prior exposure to the target environment. This mechanism enables knowledge utilization to be dynamically updated with changing observations, rather than being statically injected.

### 3.3. Knowledge-Enhanced Scene Graph Constructor

(1) Online Scene Graph Construction: To obtain an explicit structural representation of the environment, we maintain a dynamic scene graph during navigation: $G_{t}=\left(V_{t},E_{t}\right)$, where the node set $V_{t}$ represents the objects observed up to time step *t*, and the edge set $E_{t}$ represents the relations among these objects. The purpose of maintaining such an online scene graph is to convert sequential perceptual observations into a structured and continuously updated environmental memory, so that downstream decision-making is based not only on isolated visual features but also on explicit object-level and relation-level context. For each node $v_{i}\in V_{t}$, we record its visual feature, semantic category, 3D spatial information, and detection confidence, $v_{i}=\left\{v_{i}^{vis},v_{i}^{sem},v_{i}^{spa},c_{i}\right\}.$ For each edge $e_{ij}\in E_{t}$, we record its corresponding spatial or semantic relation $e_{ij}=\left\{r_{ij},w_{ij}\right\}$, where $r_{ij}$ denotes the relation type and $w_{ij}$ denotes the relation confidence. Different from simple feature stacking, our scene graph explicitly preserves both object-level and relation-level information, making it more suitable for subsequent reasoning modules. This structured representation provides a more interpretable and reasoning-friendly basis for navigation, especially when the agent needs to reason over target-related context rather than react only to the current view.

(2) Knowledge Fusion for Graph Enhancement: Since scene graphs constructed solely from online perception are easily affected by occlusion, false detections, and missing relations, we further enhance the scene graph representation with retrieved prior knowledge. The key motivation is that online perception provides grounded but incomplete evidence, whereas retrieved prior knowledge provides transferable semantic and relational regularities that can complement noisy or partial observations. Using only online perception may lead to incomplete scene graphs, while relying only on prior knowledge may weaken grounding to the current environment. By fusing the two, the agent can maintain a scene representation that is both perceptually grounded and semantically enriched. Specifically, for each node $v_{i}$, we compute attention weights according to the semantic similarity between the node representation and the relevant knowledge entries, and inject the knowledge embeddings into the node feature:

(2) $${\tilde{v}}_{i}=v_{i}+\sum_{k\in K_{t}}\alpha_{ik}k,\alpha_{ik}=\frac{\exp(\mathrm{sim}\left(v_{i},k\right)/\tau )}{\sum_{k^{\prime }\in K_{t}}\exp\left(\mathrm{sim}\left(v_{i},k^{\prime }\right)/\tau \right)}$$

This knowledge-guided update allows each node to absorb target-relevant priors in an adaptive manner, instead of uniformly injecting all retrieved knowledge into the graph. In this way, the enhanced node representation preserves current perceptual evidence while incorporating complementary prior cues that may be useful for target localization and verification. At the relation level, we further use relational knowledge to predict potentially missing edges, thereby improving the completeness of the graph structure. For any two candidate nodes $v_{i}$ and $v_{j}$, the relation prediction is formulated as ${\hat{e}}_{ij}=g_{\phi }([{\tilde{v}}_{i}∥{\tilde{v}}_{j}∥r_{ij}^{prior}])$, where $r_{ij}^{prior}$ denotes the prior relational embedding, and $g_{\phi }$ denotes the relation prediction network. Compared with a scene graph built purely from online detections, this fusion mechanism is more robust to perceptual incompleteness and more effective at recovering latent contextual structure. Through this mechanism, the proposed scene graph not only reflects “what has been observed,” but also further models “what relations may still exist.”

(3) Goal Representation and Graph Alignment: To unify the representation of different types of navigation goals, we construct a goal graph $G_{g}$ for each task (Object-Goal, Image-Instance Goal, Text-Goal) and align it with the scene graph through a cross-attention mechanism: $M_{sg}=\mathrm{CrossAttn}\left(V_{s},V_{g}\right)$, where $V_{s}$ and $V_{g}$ denote the node representations of the scene graph and the goal graph, respectively. This alignment step allows the scene graph to be filtered and reorganized according to goal-relevant cues, so that subsequent reasoning focuses on the most relevant objects, attributes, and relations under the current task. It also provides a unified interface for handling different goal modalities within the same graph-based reasoning framework. The resulting alignment is used both for target confidence estimation and for providing goal-relevant context to the subsequent reasoning module. Algorithm 1 details the construction of the knowledge-enhanced scene graph, including online graph update, prior knowledge fusion, missing-relation completion, and scene-goal alignment.
**Algorithm 1** Knowledge-Enhanced Scene Graph Construction**Require:** 
Current observation $o_{t}$, previous scene graph $G_{t-1}$, retrieved prior knowledge $K_{t}$, target description *g***Ensure:** 
Knowledge-enhanced scene graph $G_{t}=\left(V_{t},E_{t}\right)$, goal graph $G_{g}$, aligned representation $M_{sg}$  1:**Initialize:**  2:   Parse objects, attributes, and spatial cues from $o_{t}$  3:   Set $G_{t}\leftarrow G_{t-1}$  4:   5:**Step 1: Online scene graph construction**  6:**for** each detected object in $o_{t}$ **do**  7:   Extract node feature  8:   $v_{i}\leftarrow \left\{v_{i}^{vis},v_{i}^{sem},v_{i}^{spa},c_{i}\right\}$  9:   Add or update node $v_{i}$ in $V_{t}$10:**end for**11:**for** each related object pair $\left(v_{i},v_{j}\right)$ **do**12:   Estimate relation13:   $e_{ij}\leftarrow \left\{r_{ij},w_{ij}\right\}$14:   Add or update edge $e_{ij}$ in $E_{t}$15:**end for**16: 17:**Step 2: Knowledge fusion for graph enhancement**18:**for** each node $v_{i}\in V_{t}$ **do**19:   **for** each knowledge entry $k\in K_{t}$ **do**20:     $\alpha_{ik}\leftarrow \frac{\exp(\mathrm{sim}\left(v_{i},k\right)/\tau )}{\sum_{k^{\prime }\in K_{t}}\exp\left(\mathrm{sim}\left(v_{i},k^{\prime }\right)/\tau \right)}$21:   **end for**22:   ${\tilde{v}}_{i}\leftarrow v_{i}+\sum_{k\in K_{t}}\alpha_{ik}k$23:**end for**24:**for** each candidate node pair $\left(v_{i},v_{j}\right)$ **do**25:   Predict potentially missing relation26:   ${\hat{e}}_{ij}\leftarrow g_{\phi }\left([{\tilde{v}}_{i}\;∥\;{\tilde{v}}_{j}\;∥\;r_{ij}^{prior}]\right)$27:   Update $E_{t}$ with ${\hat{e}}_{ij}$ if confidence is high28:**end for**29: 30:**Step 3: Goal graph construction**31:**if** *g* is an object category **then**32:   Construct single-node goal graph $G_{g}$33:**else if** *g* is an instance image **then**34:   Parse visual attributes and construct goal graph $G_{g}$35:**else**36:   Parse object–relation structure from text and construct goal graph $G_{g}$37:**end if**38: 39:**Step 4: Scene-goal graph alignment**40:$M_{sg}\leftarrow \mathrm{CrossAttn}\left(V_{s},V_{g}\right)$41:**return** $G_{t},G_{g},M_{sg}$

### 3.4. Multi-Step Iterative Reasoning Navigator

(1) Iterative Reasoning Process: Different from traditional single-step action prediction strategies, we adopt a multi-step iterative reasoning mechanism to progressively optimize navigation decisions. The motivation is that zero-shot goal navigation usually cannot be reliably solved by a single-step score prediction, because the agent often needs to first identify plausible target-related regions, then verify candidate cues with contextual evidence, and finally execute precise goal-directed movement. A multi-step reasoning process is therefore better aligned with the progressive nature of target search in unseen environments. At each time step *t*, the navigator performs *K* reasoning iterations over the current scene graph $G_{t}$, the goal graph $G_{g}$, and the retrieved relevant knowledge $K_{t}$, so as to enable progressive decision-making from coarse-grained exploration to fine-grained target approach. Specifically, the *k*-th iteration can be formulated as

(3) $$h_{t}^{\left(k\right)}=\mathrm{Update}\left(h_{t}^{\left(k-1\right)},G_{t},G_{g},K_{t}\right),C_{t}^{\left(k\right)}=\mathrm{GenerateCandidates}\left(G_{t},h_{t}^{\left(k\right)}\right)$$

(4) $$s_{i}^{\left(k\right)}=\mathrm{Reason}\left(h_{t}^{\left(k\right)},c_{i},K_{t,i}\right),\;c_{i}\in C_{t}^{\left(k\right)}$$

where $h_{t}^{\left(k\right)}$ denotes the contextual state after the *k*-th iteration, which encodes the current environmental understanding, target semantic constraints, and historical reasoning results; $C_{t}^{\left(k\right)}$ denotes the set of candidate actions or candidate regions generated at the current stage; and $s_{i}^{\left(k\right)}$ denotes the overall score of candidate $c_{i}$. Here, Update(·) is used to integrate current observations, scene graph structure, and external knowledge to update the global contextual representation of the navigator; To improve the stability of the reasoning process, this work adopts a templated chain-of-thought prompting strategy with structured context including goal information, scene summary, retrieved knowledge, and candidate actions. GenerateCandidates(·) is used to generate a candidate decision set according to the current environmental topology, navigable regions, and goal-related cues; and Reason(·) is responsible for performing knowledge-constrained semantic reasoning and scoring for each candidate. In this way, the reasoning process is decomposed into iterative context updating, candidate generation, and candidate evaluation, which allows the policy to gradually refine its decision instead of committing to an action prematurely under incomplete observations. After *K* rounds of progressive reasoning, the navigator outputs the final action:

(5) $$a_{t}^{*}=\arg\max_{c_{i}\in C_{t}^{\left(K\right)}}s_{i}^{\left(K\right)}.$$

This coarse-to-fine reasoning process enables the agent to first conduct directional exploration and potential target region screening, and then gradually focus on local contexts relevant to the target, thereby reducing the limitations of single-step decision-making and lowering the risk of falling into local optima. In practice, we found that using a small number of reasoning steps already provides a good balance between decision quality and inference cost; therefore, *K* is treated as a tunable hyperparameter and is selected on the validation set, as discussed in Appendix A.4.

(2) Stage-adaptive Reasoning: To accommodate the requirements of different navigation stages, we follow the multi-stage navigation strategy in Unigoal [31] and adopt an adaptive stage-switching mechanism based on target confidence. The rationale for introducing stage-adaptive reasoning is that the information requirements of navigation are not constant throughout an episode. Early in navigation, the agent mainly needs broad exploration and search-space reduction; once target-related cues emerge, contextual verification becomes more important; and when the target location becomes clear, the policy should emphasize precise approach rather than broad exploration. We define the target confidence at time step *t* as

(6) $$\gamma_{t}=\max_{v_{i}\in V_{t}}\mathrm{sim}\left({\tilde{v}}_{i},v_{g}\right)\cdot c_{i}$$

where ${\tilde{v}}_{i}$ denotes the knowledge-enhanced node representation in the scene graph, $v_{g}$ denotes the target representation in the goal graph, $c_{i}$ denotes the detection confidence of the corresponding node, and sim(·,·) is the similarity function. This definition jointly considers visual–semantic matching and current perceptual reliability, and can therefore provide a relatively robust estimate of the agent’s confidence in recognizing the target. The stage transition thresholds $\theta_{1}$ and $\theta_{2}$ are selected on the validation set to balance premature switching and delayed reaction. Intuitively, $\theta_{1}$ controls when the agent should move from broad exploration to candidate verification, while $\theta_{2}$ controls when the evidence is strong enough to favor precise goal approach.

Based on the value of $\gamma_{t}$, the navigator adaptively switches among three stages. When $\gamma_{t}<\theta_{1}$, the system is in the exploration stage. At this stage, the agent still lacks sufficient evidence about the target location, and therefore prioritizes the use of semantic priors, frontier information, and environmental topology to search for potential target regions while expanding the effective observation range as much as possible. When $\theta_{1}\leq \gamma_{t}<\theta_{2}$, the system enters the verification stage. At this point, candidate cues related to the target have already emerged in the scene, and the navigator focuses on using relational knowledge, local context, and goal graph constraints to further filter candidate regions and verify their consistency. When $\gamma_{t}\geq \theta_{2}$, the system enters the approach stage. At this stage, the target location has become relatively clear, and the navigator relies more on instance-level knowledge and fine-grained visual similarity to precisely approach the target region and perform final stopping. This three-stage design is therefore consistent with the actual decision flow of goal search: semantic priors are most useful during exploration, relational reasoning is most useful during verification, and instance-level matching becomes most critical during final approach.

(3) Knowledge-aware Action Scoring: At the final action selection stage, we jointly consider the reasoning result of the language model, the strength of knowledge support, the utility of exploration, and the degree of target approach, and define the scoring function for candidate action $a_{i}$ as

(7) $$s\left(a_{i}\right)=\lambda_{1}\pi_{llm}\left(a_{i}\right)+\lambda_{2}c_{know}\left(a_{i}\right)+\lambda_{3}u_{explore}\left(a_{i}\right)+\lambda_{4}d_{goal}\left(a_{i}\right)$$

where $\pi_{llm}\left(a_{i}\right)$ denotes the preference probability assigned by the reasoning module to action $a_{i}$, which characterizes the consistency between this action and the current target-search requirement after multi-step semantic reasoning; $c_{know}\left(a_{i}\right)$ denotes the consistency between the action and prior knowledge, measuring whether the action conforms to commonsense object distribution patterns and relational constraints; $u_{explore}\left(a_{i}\right)$ denotes the potential information gain brought by the action, encouraging the agent to prioritize actions that can expand the effective observation range or reduce environmental uncertainty; and $d_{goal}\left(a_{i}\right)$ denotes an estimated goal-approach score, measuring whether action $a_{i}$ is expected to move the agent closer to the current target hypothesis maintained by the goal-aligned scene representation. It is computed solely from online observations, the maintained scene representation, and the current goal belief, without using ground-truth target coordinates, shortest-path distance, or any other oracle signals at test time. $\lambda_{1},\lambda_{2},\lambda_{3},$ and $\lambda_{4}$ are the weighting coefficients used to balance the influence of different factors on the final decision. The weighting coefficients are determined through validation-based tuning to balance semantic reasoning, prior consistency, exploration efficiency, and goal-directed movement. In particular, larger $\lambda_{1}$ emphasizes the reasoning module, whereas larger $\lambda_{3}$ encourages exploratory behavior; the final setting is chosen to provide stable performance across different goal modalities rather than over-optimizing a single task. Algorithm 2 shows the overall online navigation procedure of PriorNav. At each time step, the agent first updates a knowledge-enhanced scene graph and estimates the target confidence, and then performs stage-adaptive multi-step reasoning to produce the final navigation action.
**Algorithm 2** Stage-Adaptive Multi-step Iterative Reasoning for PriorNav**Require:** 
Current observation $o_{t}$, target description *g*, previous scene graph $G_{t-1}$, prior knowledge base K, reasoning iteration number *K*, stage thresholds $\theta_{1},\theta_{2}$, scoring weights $\lambda_{1},\lambda_{2},\lambda_{3},\lambda_{4}$**Ensure:** 
Final navigation action $a_{t}^{*}$  1:**Initialize:**  2:   Construct query representation $q_{t}$ from $o_{t}$ and *g*  3:   Retrieve relevant prior knowledge $K_{t}\leftarrow \mathrm{TopK}\left(\mathrm{sim}(q_{t},k_{i})\right)$ from K  4:   Update scene graph $G_{t}=\left(V_{t},E_{t}\right)$ from $o_{t}$ and $G_{t-1}$  5:   Construct goal graph $G_{g}$ from *g*  6:   7:**Step 1: Knowledge-enhanced scene graph construction**  8:**for** each node $v_{i}\in V_{t}$ **do**  9:   Compute attention weights10:   $\alpha_{ik}\leftarrow \frac{\exp(\mathrm{sim}\left(v_{i},k\right)/\tau )}{\sum_{k^{\prime }\in K_{t}}\exp\left(\mathrm{sim}\left(v_{i},k^{\prime }\right)/\tau \right)}$11:   Fuse prior knowledge into node feature12:   ${\tilde{v}}_{i}\leftarrow v_{i}+\sum_{k\in K_{t}}\alpha_{ik}k$13:**end for**14:**for** each candidate node pair $\left(v_{i},v_{j}\right)$ **do**15:   Predict missing relation16:   ${\hat{e}}_{ij}\leftarrow g_{\phi }\left([{\tilde{v}}_{i}\;∥\;{\tilde{v}}_{j}\;∥\;r_{ij}^{prior}]\right)$17:**end for**18: 19:**Step 2: Goal alignment and target confidence estimation**20:$M_{sg}\leftarrow \mathrm{CrossAttn}\left(V_{s},V_{g}\right)$21:$\gamma_{t}\leftarrow \max_{v_{i}\in V_{t}}\mathrm{sim}\left({\tilde{v}}_{i},v_{g}\right)\cdot c_{i}$22: 23:**Step 3: Stage-adaptive switching**24:**if** $\gamma_{t}<\theta_{1}$ **then**25:   stage ← Exploration26:**else if** *θ*_1_ ≤ *γ*_*t*_ < *θ*_2_ **then**27:   stage ← Verification28:**else**29:   stage ← Approach30:**end if**31: 32:**Step 4: Multi-step iterative reasoning**33:Initialize contextual state $h_{t}^{\left(0\right)}$ from $G_{t}$, $G_{g}$, $K_{t}$, and $M_{sg}$34:**for** k=1 **to** *K* **do**35:   Update contextual state36:   $h_{t}^{\left(k\right)}\leftarrow \mathrm{Update}\;\left(h_{t}^{\left(k-1\right)},G_{t},G_{g},K_{t}\right)$37:   Generate stage-specific candidates38:   $C_{t}^{\left(k\right)}\leftarrow \mathrm{GenerateCandidates}\;\left(G_{t},h_{t}^{\left(k\right)}\right)$39:   **for** each candidate $c_{i}\in C_{t}^{\left(k\right)}$ **do**40:       Score candidate by knowledge-constrained reasoning41:       $s_{i}^{\left(k\right)}\leftarrow \mathrm{Reason}\;\left(h_{t}^{\left(k\right)},c_{i},K_{t,i}\right)$42:   **end for**43:**end for**44: 45:**Step 5: Knowledge-aware action scoring**46:**for** each candidate action $a_{i}\in C_{t}^{\left(K\right)}$ **do**47:   $s\left(a_{i}\right)\leftarrow \lambda_{1}\pi_{\mathrm{llm}}\left(a_{i}\right)+\lambda_{2}c_{\mathrm{know}}\left(a_{i}\right)+\lambda_{3}u_{\mathrm{explore}}\left(a_{i}\right)+\lambda_{4}d_{\mathrm{goal}}\left(a_{i}\right)$48:**end for**49: 50:**Step 6: Final action selection**51:$a_{t}^{*}\leftarrow \arg\max_{a_{i}\in C_{t}^{\left(K\right)}}s\left(a_{i}\right)$52:**return** $a_{t}^{*}$

## 4. Experiments

In this section, we conduct a comprehensive evaluation of the effectiveness of PriorNav on zero-shot goal navigation tasks. We first present the implementation and experimental setup, including task definitions, datasets, evaluation metrics, and implementation details. We then report the main results by comparing PriorNav with state-of-the-art methods, followed by extensive ablation studies to analyze the contribution of each component in our framework.

### 4.1. Experimental Setup

We evaluate PriorNav on three zero-shot navigation tasks: Object-Goal, Image-Instance Goal, and Text-Goal navigation. The experiments are conducted on the Habitat-Matterport3D (HM3D) dataset [33] following the standard train/test split, with zero-shot cross-dataset evaluation performed on Matterport3D (MP3D) [2]. We adopt Success Rate (SR) and Success weighted by Path Length (SPL) as the primary evaluation metrics. Detailed task definitions, dataset statistics, evaluation protocols, and implementation configurations are provided in Appendix A.

### 4.2. Main Results

#### 4.2.1. Comparison with State-of-the-Art Methods

Baseline: We select the baselines in Table 1 to provide a balanced comparison from three perspectives: task coverage, training paradigm, and reasoning mechanism. First, we include representative task-specific baselines, including SemExp [34], ZSON [25], OVRL-v2 [35], Krantz et al. [36], OVRL-v2-IIN [35], and IEVE [37], which cover Object-Goal or Image-Instance Goal navigation under semantic exploration, multimodal goal embedding, and instance-level localization settings. These methods provide strong references for evaluating whether PriorNav improves over earlier semantic-navigation and instance-navigation pipelines. Second, we include universal baselines, including PSL [38], GOAT [39], and UniGoal [31], because our goal is not only to improve task-specific performance but also to evaluate whether PriorNav remains competitive under a unified multi-goal setting. In particular, UniGoal [31] is the most directly related baseline, since it adopts a unified graph representation to jointly support Object-Goal, Image-Instance Goal, and Text-Goal navigation within a single zero-shot framework. Third, we include recent training-free and foundation-model-driven methods, including ESC [22], OpenFMNav [28], VLFM [29], SG-Nav [30], and Mod-IIN [40], to compare PriorNav against methods that rely on commonsense constraints, vision-language similarity, scene-graph prompting, or explicit large-model reasoning rather than task-specific policy training. This baseline set therefore covers both classical and recent methods, both task-specific and universal methods, and both trained and training-free zero-shot navigation paradigms. We note that some methods are reported only on a subset of tasks because they were originally designed for a specific goal modality or were not evaluated under all corresponding benchmark protocols in their original papers.

Performance Comparison on the Test Set: As shown in Table 1, PriorNav achieves the best overall performance across the three zero-shot goal navigation tasks, while maintaining consistent gains in both success rate and path efficiency. Compared with the strongest reported baseline under each corresponding task setting, our method improves success rate by 3.5%, 13.3%, and 3.5% on Object-Goal, Image-Instance Goal, and Text-Goal navigation, respectively. These results indicate that jointly integrating multi-level prior knowledge, knowledge-enhanced scene representation, and multi-step iterative reasoning leads to more reliable target search in unseen environments.

Compared with task-specific baselines and recent training-free methods, PriorNav shows stronger and more consistent performance across different goal modalities. In particular, relative to VLFM and SG-Nav on Object-Goal navigation, the improvement suggests that relying only on vision-language similarity or online graph prompting is still insufficient for robust target search in unseen scenes, whereas the introduction of retrievable prior knowledge and stage-adaptive reasoning provides stronger guidance for search-space reduction and decision refinement. Compared with UniGoal, which is the most directly related universal baseline, PriorNav further demonstrates that a unified graph representation can be substantially strengthened by explicitly incorporating semantic, instance-level, and relational priors into the decision process rather than depending mainly on graph matching and multi-stage exploration alone.

From a task-specific perspective, the gain is most pronounced on the Image-Instance Goal task, where accurate instance-level discrimination is especially important. This result suggests that the combination of instance knowledge and knowledge-enhanced scene graph construction is particularly beneficial for fine-grained target verification and disambiguation. On the Text-Goal task, PriorNav also yields clear improvements, indicating that the proposed framework is effective in handling richer semantic constraints and multi-attribute target descriptions, where relational knowledge and iterative reasoning play a more important role. On the Object-Goal task, the performance gain further confirms that semantic priors and structured reasoning help the agent narrow the search space and reach plausible target regions more efficiently.

#### 4.2.2. Ablation Studies

**Figure 2:**
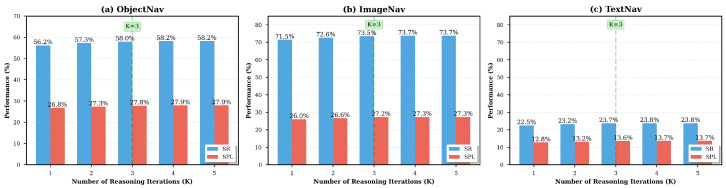
Impact of reasoning iterations *K* on navigation performance.

To further understand why PriorNav improves zero-shot goal navigation, we conduct ablation studies from three perspectives: multi-level prior knowledge, multi-step iterative reasoning, and knowledge-enhanced scene graph construction. Algorithms 1 and 2 are evaluated as functional modules within the full PriorNav framework rather than as standalone tasks. Specifically, Algorithm 1 is validated through the ablation study of the knowledge-enhanced scene graph in Table 2, which isolates the contribution of structured scene-graph construction and prior-knowledge enhancement. Algorithm 2 is validated through both the reasoning-step analysis in Figure 2 and the overall navigation performance in Table 3, since it represents the complete online decision-making procedure of PriorNav rather than a single isolated component. These experiments are designed to separately evaluate the contribution of each major component in the proposed framework and to reveal how different design choices affect different goal modalities.

Prior Knowledge: We first study the contribution of different prior knowledge types by selectively removing each knowledge level from the full model. The results are reported in Table 3. This ablation directly evaluates whether semantic, instance-level, and relational priors provide complementary guidance during navigation, rather than merely acting as redundant auxiliary information. All three knowledge levels contribute to the final performance, and removing any single type leads to a decrease in navigation performance, confirming that multi-level prior knowledge provides complementary information for target search and decision-making.

Semantic knowledge has the largest influence overall. Removing semantic knowledge causes the most notable drop on both ON-SR and TN-SR, indicating that semantic priors provide the primary guidance for identifying plausible target regions and supporting goal-oriented exploration. This observation is consistent with the role of semantic knowledge in our framework: it offers category-level regularities about likely target locations and co-occurring context, which are especially important when the agent must reduce the search space in unseen scenes. This effect is particularly evident in the TN task, where semantic knowledge is important for interpreting textual descriptions and aligning language constraints with the scene context.

Relational knowledge shows a more visible contribution in the IIN and TN tasks. The corresponding performance drop after removing relational knowledge suggests that spatial and contextual relations provide useful complementary cues for target localization. In the IIN task, such relations help the agent verify instance-level targets using surrounding context, while in the TN task they support the interpretation of compositional descriptions involving multiple objects or attributes. This result further indicates that relational priors are particularly useful when successful navigation depends not only on target appearance but also on understanding how the target is situated within a larger scene context.

Instance knowledge also contributes consistently, especially when target discrimination depends on appearance-level cues. Removing instance knowledge leads to performance drops on all three tasks, indicating that instance-level representations help improve fine-grained matching between the target and current observations. Its contribution is relatively more apparent in tasks that require distinguishing visually similar objects, while in the TN task its role is comparatively weaker because textual descriptions are often dominated by semantic and relational constraints. Overall, Table 3 verifies that the three knowledge levels are not interchangeable: semantic knowledge mainly supports search-space reduction, instance knowledge mainly supports fine-grained matching, and relational knowledge mainly supports context-based verification.

Multi-step Iterative Reasoning: We next analyze the contribution of iterative reasoning by varying the number of reasoning iterations *K*, as shown in Figure 2. This experiment evaluates whether the performance gain of PriorNav comes only from richer scene representation, or whether progressively refining decisions through multi-step reasoning provides an additional benefit. Setting K=3 achieves the best trade-off between performance and efficiency, yielding clear improvements over single-step reasoning (+1.8% ON-SR, +2.0% IIN-SR, and +1.2% TN-SR) while maintaining a practical inference cost (213 ms/step). Further increasing *K* brings only minor additional gains, indicating that a moderate number of reasoning steps is sufficient to capture most of the benefit of iterative reasoning in the current framework.

From a task-specific perspective, the IIN task benefits the most from iterative reasoning, suggesting that multi-step reasoning is particularly helpful for instance-level target verification and contextual disambiguation. This is reasonable because image-instance navigation often requires the agent to iteratively compare visual candidates, reject distracting but similar objects, and refine its belief using surrounding context. The ON and TN tasks also show consistent improvements as *K* increases, which confirms that iterative reasoning contributes positively across different goal modalities. Overall, these results show that multi-step reasoning improves navigation decisions by progressively refining action selection, while a small number of reasoning steps is already effective in balancing accuracy and efficiency. Although increasing the number of reasoning steps can further improve decision quality, the marginal gain becomes limited beyond K=3, while latency continues to increase. Therefore, K=3 is adopted as the default setting to balance navigation accuracy and online decision efficiency. This ablation directly evaluates the effectiveness of Algorithm 1 by isolating the contribution of scene-graph construction and prior-knowledge enhancement within the full navigation framework. Together with the main comparison results in Table 1, this analysis justifies the effectiveness of Algorithm 2 as the complete online decision-making procedure of PriorNav.

Knowledge-enhanced Scene Graph: Finally, we evaluate the contribution of structured scene modeling and knowledge enhancement, as reported in Table 2. This ablation is designed to distinguish the benefit of using a scene graph itself from the additional benefit of injecting retrieved prior knowledge into that graph. Removing knowledge enhancement from the scene graph leads to consistent performance drops on all three tasks (−1.7% ON-SR, −1.3% IIN-SR, and −1.6% TN-SR), showing that integrating prior knowledge with online observations provides useful additional cues for navigation and target localization.

From a task-specific perspective, the effect of removing knowledge enhancement is slightly more pronounced on the TN task and remains comparable on the ON task, while the drop on the IIN task is relatively smaller. This suggests that knowledge enhancement is especially helpful when the agent needs to interpret semantic relations and contextual constraints beyond direct visual matching. In the TN task, textual descriptions often involve abstract semantic relations or compositional constraints, for which knowledge enhancement helps complete the scene understanding process. In contrast, in the IIN task, the target image already provides richer visual guidance, so the relative contribution of knowledge enhancement is somewhat smaller.

Even without knowledge enhancement, the scene graph alone still brings clear improvements over the variant without a scene graph (w/o Scene Graph), with gains of about 1.8% ON-SR, 2.4% IIN-SR, and 1.6% TN-SR. This comparison shows that the scene graph itself already provides a useful structured memory for organizing online observations, while knowledge enhancement further strengthens this representation by supplying missing semantic and relational cues. This further confirms the value of structured scene representation for zero-shot goal navigation. Overall, the results show that scene-graph construction and knowledge enhancement play complementary roles: the former organizes online observations into a structured representation, while the latter enriches this representation with additional semantic and relational cues.

#### 4.2.3. Computational Cost and Efficiency Analysis

Beyond navigation accuracy, computational efficiency is also important for assessing the practical applicability of PriorNav. Since our framework integrates prior knowledge retrieval, online scene-graph construction, and multi-step reasoning with a locally deployed large language model, it incurs additional inference overhead compared with simpler similarity-based or single-stage navigation pipelines. This subsection analyzes the computational characteristics of the proposed framework and explains the rationale behind key design choices.

All experiments are conducted on a platform equipped with an NVIDIA RTX 4090 GPU. Under the default setting K=3, PriorNav requires approximately 213 ms per navigation step on average. The reported per-step latency is measured over online inference during evaluation episodes under the default setting K=3, and includes prior knowledge retrieval, knowledge-enhanced scene graph update, iterative reasoning, and final action scoring. The total latency is measured directly from the deployed system, while the component-wise percentages in Table 4 are provided as approximate interpretation-oriented estimates rather than strictly profiler-derived numbers. To provide insight into how this overall latency is distributed across components, Table 4 presents an approximate discussion-level breakdown. The multi-step reasoning module constitutes the dominant computational burden, accounting for approximately 55–65% of the total runtime. This is consistent with the architectural design, as each of the three reasoning iterations involves forward passes through the LLaMA-2-7B model, which serves as the reasoning backbone.

The knowledge-enhanced scene graph update represents a moderate portion of the runtime, estimated at approximately 15–20%. This module involves continuous graph construction, relation modeling, and the integration of retrieved prior knowledge into the evolving scene representation at each step. The computational footprint remains moderate due to the use of an efficient graph-based representation and incremental update mechanisms. Prior knowledge retrieval contributes an estimated 8–12% of the latency, implemented via vector similarity search over indexed prior knowledge bases. Goal-aware action scoring accounts for a similar fraction, involving lightweight computation of compatibility scores between candidate actions and the reasoning output.

The total per-step latency of 213 ms is measured experimentally. The component-wise distribution represents a coarse breakdown estimated for interpretation and discussion, rather than precisely profiled module-wise timings.

The computational cost of PriorNav is directly influenced by the number of reasoning steps *K*. Increasing *K* allows more progressive refinement of candidate actions through deeper multi-step reasoning, since each iteration provides an opportunity to correct earlier mispredictions and incorporate updated scene-context information. However, additional reasoning steps also introduce noticeable runtime overhead. As shown in Figure 2, K=3 provides the best trade-off between navigation performance and runtime efficiency in our setting. At smaller values (K=1 or K=2), the reasoning process has fewer opportunities to refine decisions, which limits the effectiveness of prior knowledge integration and contextual reasoning. At larger values (K≥4), the marginal benefits of additional reasoning diminish while latency increases substantially. Therefore, we adopt K=3 as the default setting across all experiments.

PriorNav employs a stage-adaptive reasoning strategy that dynamically adjusts navigation behavior according to the accumulated evidence about the target location. This strategy partitions the navigation process into three distinct phases: exploration, verification, and approach. The transitions between these stages are controlled by two confidence thresholds, denoted as $\theta_{1}$ and $\theta_{2}$ with $\theta_{1}<\theta_{2}$.

The threshold $\theta_{1}$ controls the transition from broad exploration to candidate verification. When the maximum target confidence across all candidate objects remains below $\theta_{1}$, the agent maintains exploratory behavior and considers a wide set of candidate actions for systematic scene coverage. Once the confidence exceeds $\theta_{1}$, the system enters the verification stage, where reasoning becomes more selective and focuses on contextual verification of promising candidate locations. The threshold $\theta_{2}$ controls when the accumulated evidence becomes strong enough to favor precise goal-oriented approach. When confidence exceeds $\theta_{2}$, the agent prioritizes precise movement toward the most likely target location, thereby reducing unnecessary deviations and path-length inefficiency.

These thresholds are selected on the validation set to balance two competing risks: premature switching, where the system commits to an incorrect target before sufficient evidence is accumulated, and delayed reaction, where the system continues exploratory behavior even after the target location has been identified with high confidence. The three-stage design therefore helps PriorNav organize reasoning in a more focused manner, with broader reasoning during early exploration and increasingly selective reasoning as confidence accumulates.

#### 4.2.4. Limitations and Discussion

Although PriorNav achieves consistent improvements across multiple zero-shot goal navigation tasks, the current framework still has several limitations. First, all evaluations in this work are conducted in simulation environments, and real-world robotic deployment is not yet included. As a result, the practical performance of PriorNav may still be affected by sim-to-real gaps such as sensor noise, actuation errors, imperfect object detection, and limited onboard computing resources. Therefore, our current conclusions should be interpreted as demonstrating strong effectiveness in realistic simulation benchmarks rather than complete readiness for physical deployment.

The current framework may still encounter difficulties in highly dynamic scenes or in environments containing many visually similar objects. In dynamic environments, rapid object changes may reduce the stability of the maintained scene graph and weaken the reliability of relation modeling. In addition, when multiple candidate objects share highly similar visual appearance or semantic context, instance-level discrimination and target verification become more challenging, even with the support of prior knowledge and iterative reasoning.

Although the multi-step reasoning design improves navigation decisions, it also increases computational complexity and makes the method less suitable for highly resource-constrained platforms. As discussed in the previous subsection, the use of a locally deployed LLaMA-2-7B and a dynamically updated knowledge-enhanced scene graph introduces additional runtime overhead. This trade-off is acceptable in our current experimental setting, but further optimization is still necessary for long-horizon deployment and real-time embodied applications.

Despite these limitations, the proposed framework provides a useful step toward more reliable zero-shot goal navigation by explicitly integrating transferable prior knowledge, structured scene representation, and progressive reasoning within a unified pipeline. In future work, we plan to investigate lightweight reasoning architectures, stronger handling of dynamic scenes, and real-world robotic validation to further improve the practicality and generality of the framework.

## 5. Conclusions

In zero-shot goal navigation, relying solely on online observations and local matching is insufficient for complex target search in unseen environments. To address this limitation, we propose PriorNav, a prior-knowledge-enhanced framework that jointly models multi-level prior knowledge, a knowledge-enhanced scene graph, and multi-step iterative reasoning. By integrating structured prior knowledge with explicit scene representation and progressive reasoning, PriorNav enables more reliable decision-making across exploration, verification, and approach stages.

Experiments on Object-Goal, Image-Instance Goal, and Text-Goal navigation show that PriorNav achieves consistent improvements over strong baselines, with clearer advantages in scenarios requiring richer contextual understanding and semantic reasoning. Ablation studies further indicate that these gains come from the synergy among prior knowledge, structured representation, and iterative reasoning. Future work will focus on online knowledge updating, more robust scene relation modeling, and lightweight reasoning mechanisms for more open-ended and real-world navigation tasks. 

## Figures and Tables

**Table 1:** Performance comparison across different zero-shot navigation tasks and benchmarks. The selected baselines cover task-specific, universal, and training-free/foundation-model-driven methods.

Method	Training-Free	Universal	ON				IIN		TN	
			MP3D		HM3D		HM3D		HM3D	
			SR	SPL	SR	SPL	SR	SPL	SR	SPL
SemExp [34]	×	×	36.0	14.4	–	–	–	–	–	–
ZSON [25]	×	×	15.3	4.8	25.5	12.6	–	–	–	–
OVRL-v2 [35]	×	×	–	–	64.7	28.1	–	–	–	–
Krantz et al. [36]	×	×	–	–	–	–	8.3	3.5	–	–
OVRL-v2-IIN [35]	×	×	–	–	–	–	24.8	11.8	–	–
IEVE [37]	×	×	–	–	–	–	70.2	25.2	–	–
PSL [38]	×	✓	–	–	42.4	19.2	23.0	11.4	16.5	7.5
GOAT [39]	×	✓	–	–	50.6	24.1	37.4	16.1	17.0	8.8
ESC [22]	✓	×	28.7	14.2	39.2	22.3	–	–	–	–
OpenFMNav [28]	✓	×	37.2	15.7	52.5	24.1	–	–	–	–
VLFM [29]	✓	×	36.2	15.9	52.4	**30.3**	–	–	–	–
SG-Nav [30]	✓	×	40.2	16.0	54.0	24.9	–	–	–	–
Mod-IIN [40]	✓	×	–	–	–	–	56.1	23.3	–	–
UniGoal [31]	✓	✓	41.0	16.4	54.5	25.1	60.2	23.7	20.2	11.4
PriorNav	✓	✓	**44.3**	**19.2**	**58.0**	27.8	**73.5**	**27.2**	**23.7**	**13.6**

**Note:** ON, IIN, and TN denote Object-Goal Navigation, Image-Instance Navigation, and Text-Goal Navigation, respectively. “Training-Free” indicates methods that do not require task-specific policy training for the target benchmark, and “Universal” indicates methods designed to support multiple goal modalities within a unified framework. ✓ indicates that the corresponding property is satisfied, whereas × indicates that the corresponding property is not satisfied. Bold values indicate the best performance in each column. “–” means that the corresponding result was not reported in the original paper or the method was not evaluated under that benchmark/task setting.

**Table 2:** Ablation study of the knowledge-enhanced scene graph on HM3D.

Variant	Scene Graph	Knowledge Enh.	ON		IIN		TN	
			SR	SPL	SR	SPL	SR	SPL
PriorNav (full)	✓	✓	**58.0%**	**27.8%**	**73.5%**	**27.2%**	**23.7%**	**13.6%**
w/o Knowledge Enh.	✓	×	56.3%	26.6%	72.2%	26.5%	22.1%	12.7%
w/o Scene Graph	×	×	54.5%	25.5%	69.8%	25.2%	20.5%	11.8%

**Table 3:** Ablation study of prior knowledge components on HM3D.

Variant	Semantic	Instance	Relational	ON		IIN		TN	
				SR	SPL	SR	SPL	SR	SPL
PriorNav (full)	✓	✓	✓	**58.0%**	**27.8%**	**73.5%**	**27.2%**	**23.7%**	**13.6%**
w/o Semantic	×	✓	✓	55.8%	26.5%	71.9%	26.3%	21.5%	12.3%
w/o Instance	✓	×	✓	56.7%	27.2%	72.6%	26.9%	22.8%	13.2%
w/o Relational	✓	✓	×	57.0%	27.4%	73.0%	27.0%	23.2%	13.3%
w/o Knowledge (all)	×	×	×	54.5%	25.5%	69.8%	25.2%	20.5%	11.8%

**Table 4:** Approximate component-wise runtime allocation per navigation step (K=3).

Component	Est. Time (ms)	Est. Contribution	Computational Nature
Prior knowledge retrieval	∼20	∼9%	Vector similarity search
Knowledge-enhanced scene graph update	∼35	∼16%	Incremental graph construction
Multi-step iterative reasoning (K=3)	∼130	∼61%	3 × LLaMA-2-7B forward passes
Goal-aware action scoring	∼23	∼11%	Compatibility computation
System overhead	∼5	∼2%	Data transfer, synchronization

**Figure A1:**
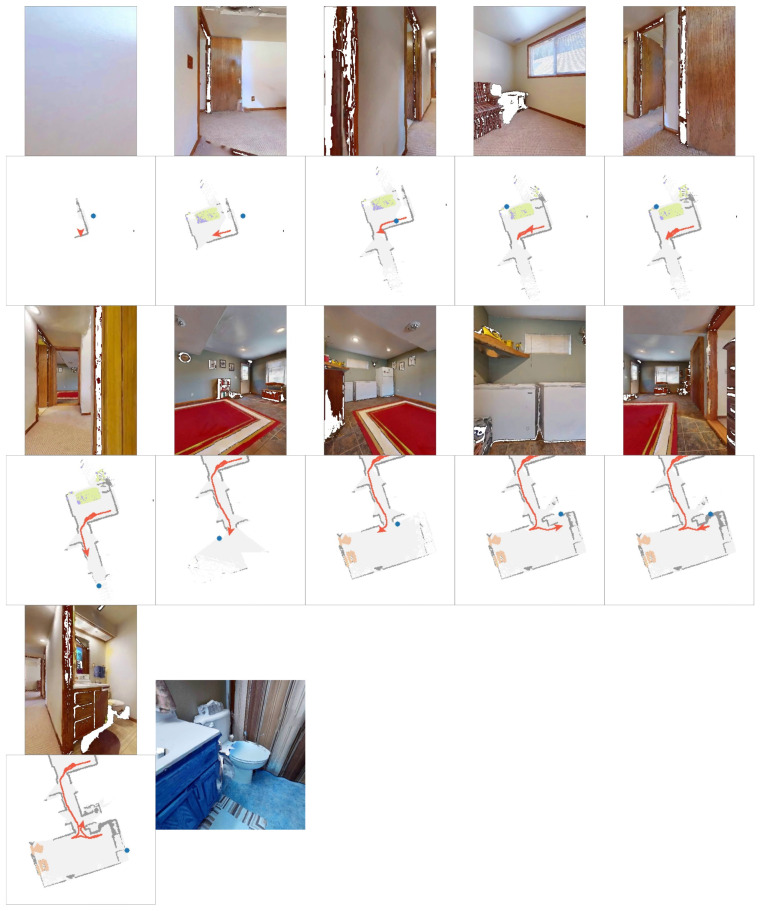
Qualitative Success Case.

**Figure A2:**
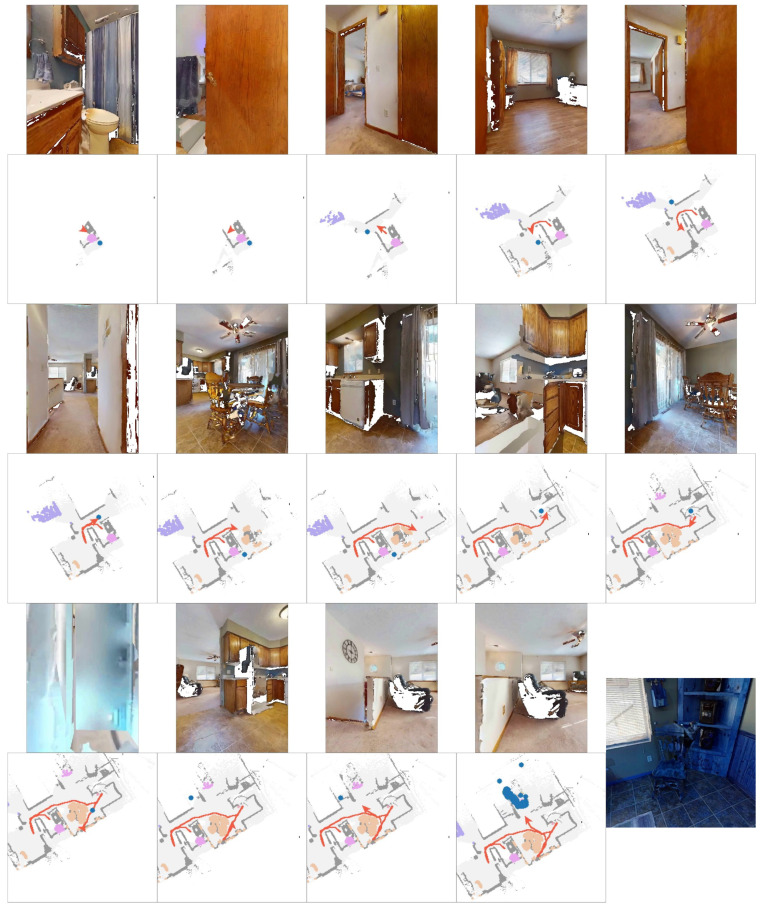
Qualitative Failure Case.

## Data Availability

The data supporting the reported results in this study are from publicly available datasets, including HM3D and MP3D. Access to and use of these datasets are subject to their respective data license agreements, access policies, and usage restrictions. No new datasets were created in this study.

## Appendix A. Experimental Setup

### Appendix A.1. Task Definitions

We evaluate PriorNav on three zero-shot goal navigation sub-tasks, each of which involves different goal modalities and levels of complexity.

Object-Goal Navigation (ON): Given an object category (e.g., “chair”), the agent must navigate to any instance of that category in an unseen environment. The agent receives the object category as a textual label and must explore the environment to locate and reach a position within 1 m of any instance of the specified object. This task evaluates the agent’s ability to leverage semantic knowledge about typical object locations and co-occurrence patterns.

Image-Instance Navigation (IIN): Given a reference image depicting a specific object instance, the agent must find that exact instance in the environment. The target image contains both the visual appearance of the target object and the surrounding contextual cues, requiring fine-grained visual matching and spatial reasoning. This task evaluates the agent’s ability in instance-level knowledge integration and visual localization.

Text-Goal Navigation (TN): Given a rich textual description of the target (e.g., “Navigate to the bedroom with a white bed and two pillows”), the agent must locate the target that satisfies all specified constraints. This task is the most challenging, as it requires deep language understanding, multi-attribute reasoning, and complex spatial relation comprehension. It particularly tests the agent’s ability to leverage relational knowledge and perform multi-step logical reasoning.

For all three tasks, the agent starts from a random position in an unseen environment and must decide when to stop based on its observations and reasoning. The maximum episode length is set to 500 steps to ensure sufficient exploration time while preventing infinite loops.

### Appendix A.2. Dataset Details

We conduct experiments on two large-scale real-world indoor environment datasets:

Habitat-Matterport3D (HM3D): HM3D [33] is a large-scale benchmark for embodied navigation in indoor environments, providing photorealistic 3D scene meshes and panoramic RGB-D observations. Following the standard split, we use 72 scenes for training and 18 scenes for testing. Unless otherwise specified, all models are trained exclusively on the HM3D training split and evaluated on previously unseen test scenes.

Matterport3D (MP3D): This dataset [2] contains indoor scenes from 90 buildings, and we use it for zero-shot cross-dataset generalization evaluation. No training data from MP3D is used during model training, making this a strict zero-shot transfer setting.

### Appendix A.3. Evaluation Metrics

We adopt the standard evaluation metrics for object navigation:

Success Rate (SR): The percentage of episodes in which the agent successfully reaches within 1 m Euclidean distance of the target object. SR measures the overall navigation effectiveness.

Success weighted by Path Length (SPL): SPL jointly considers success rate and path efficiency, and is defined as

(A1) $$SPL=\frac{1}{N}\sum_{i=1}^{N}\frac{S_{i}\cdot l_{i}}{\max(l_{i},p_{i})}$$

where $S_{i}\in \left\{0,1\right\}$ indicates whether episode *i* is successful, $l_{i}$ is the shortest path length to the target, and $p_{i}$ is the actual path length taken by the agent. SPL rewards efficient trajectories that are close to the optimal path.

Navigation Error (NE): The average Euclidean distance (in meters) between the agent’s final position and the target position.

### Appendix A.4. Implementation Configuration

#### Appendix A.4.1. Knowledge Base Construction

We build a comprehensive prior knowledge base from multiple heterogeneous sources. Semantic knowledge is extracted from WordNet [4], which provides object categories and semantic hierarchies, and ConceptNet [5], which provides commonsense associations and relational triplets. This knowledge includes typical object locations, co-occurrence patterns, and functional attributes. Instance knowledge is obtained from visual datasets and image-based feature extraction pipelines, where object appearance cues such as color, material, local texture, and spatial extent are encoded into visual–semantic representations. Relational knowledge is derived from Visual Genome [6] and from spatial statistics collected from the HM3D training split, including spatial relations such as adjacency, support, and relative position, as well as typical room-layout regularities.

To organize these heterogeneous sources into a unified knowledge base, we divide the extracted entries into three levels: semantic knowledge, instance knowledge, and relational knowledge. Semantic knowledge includes typical locations, co-occurring entities, and functional descriptions of target categories. Instance knowledge includes appearance-related cues such as attributes, materials, colors, and local visual descriptions. Relational knowledge includes spatial and semantic relations between object pairs, such as support, adjacency, containment, and typical contextual dependencies.

For each knowledge entry, we first convert the original symbolic or textual representation into a textual template. These templates are then embedded into a shared vector space through contrastive representation learning, so that knowledge items from different sources can be retrieved within the same similarity-based framework. In this way, the resulting prior knowledge base supports target-aware and scene-aware retrieval across multiple knowledge types.

#### Appendix A.4.2. Knowledge Embedding and Retrieval

All prior knowledge entries are encoded into a unified embedding space and indexed for efficient retrieval. During navigation, the agent constructs a query representation from the current observation, goal specification, and scene context, and performs nearest-neighbor search over the indexed knowledge base to retrieve the most relevant semantic, instance-level, and relational priors.

In our implementation, the vector index is built using FAISS, which enables efficient similarity-based retrieval over a large number of prior knowledge entries. The retrieved results are then filtered by relevance and passed to the downstream scene-graph enhancement and reasoning modules. The retrieval mechanism is dynamic: as the observation and target belief evolve during navigation, the query representation is updated accordingly, and the retrieved knowledge is refreshed at each step rather than being statically injected once at the beginning of the episode.

Given a knowledge item $k_{i}$, its embedding is defined as $k_{i}=f_{\theta }\left(k_{i}\right)$, where $f_{\theta }\left(\cdot \right)$ denotes the knowledge encoder. All knowledge is encoded into a shared embedding space through contrastive learning, so that semantic descriptions, visual attributes, and relational texts can be aligned under a unified retrieval framework.

#### Appendix A.4.3. Network Architecture

The visual encoder is a ResNet-50 pretrained on ImageNet [41,42] and frozen during training. It extracts 2048-dimensional features, which are projected into 512-dimensional embeddings through two MLP layers.

The scene graph encoder is a 3-layer graph attention network (GAT) [43] with 8 attention heads, a hidden dimension of 512, GELU activation, and a dropout rate of 0.1. Each node in the scene graph is represented as $v_{i}=\left\{v_{i}^{vis},v_{i}^{sem},v_{i}^{spa},c_{i}\right\}$, where $v_{i}^{vis}$ denotes visual features, $v_{i}^{sem}$ denotes semantic embeddings, $v_{i}^{spa}$ denotes 3D spatial coordinates, and $c_{i}$ denotes detection confidence. Each edge is represented as $e_{ij}=\left\{r_{ij},w_{ij}\right\}$, where $r_{ij}$ denotes the relation type and $w_{ij}$ denotes the relation confidence.

The LLM reasoning module is based on LLaMA-2-7B [44], using templated chain-of-thought prompting with a maximum context length of 2048 tokens. The large language model is locally deployed as an inference module for navigation decision-making. At each time step, the system compresses the current knowledge-enhanced scene graph, goal graph, and retrieved prior knowledge into a fixed-format structured context, which is then fed into LLaMA-2-7B for forward inference. This process neither depends on cloud services nor involves open-ended multi-turn textual dialogue, thereby ensuring experimental reproducibility and controllable inference latency.

The policy network is implemented as a 6-layer Transformer encoder with 8 attention heads and a hidden dimension of 512. It first estimates a score distribution over candidate navigable directions, and then converts the selected direction into a discrete control action.

#### Appendix A.4.4. Training Strategy

Considering that knowledge retrieval, scene graph construction, and large language model reasoning all involve discrete or non-differentiable processes, we adopt a stage-wise training strategy. First, the prior knowledge base is constructed offline and a unified knowledge embedding space is learned. Then, the knowledge-enhanced scene graph encoder and the navigation policy network are trained. During inference, the large language model serves as a frozen semantic reasoner to score candidate actions and is not involved in gradient updates.

Stage 1: We first train the scene graph encoder on HM3D with a batch size of 64 and a learning rate of $1\times 10^{-4}$.

Stage 2: During knowledge fusion, the scene graph encoder is frozen and only the knowledge attention module is trained with a learning rate of $5\times 10^{-5}$.

Stage 3: The full model is then trained end-to-end with a learning rate of $1\times 10^{-5}$.

The multi-task loss function is defined as

(A2) $$L=L_{nav}+0.5L_{know}+0.3L_{align}+0.2L_{smooth},$$

where $L_{nav}$ denotes the navigation cross-entropy loss, $L_{know}$ denotes the knowledge consistency loss, $L_{align}$ denotes the scene-goal alignment loss, and $L_{smooth}$ denotes the action smoothing loss.

#### Appendix A.4.5. Inference Configuration and Hyperparameter Selection

Unless otherwise specified, all main experiments use the same inference configuration. The number of reasoning iterations is selected from K∈{1,2,3,4,5} based on validation-set performance. We choose K=3 as the default setting because it provides the best trade-off between navigation effectiveness and inference cost in the current framework.

The stage transition thresholds $\theta_{1}$ and $\theta_{2}$ are also selected on the validation set. Their role is to control the transition from broad exploration to candidate verification and from verification to precise target approach, respectively. In practice, these thresholds are tuned to balance two competing failure modes: premature switching, where the agent commits too early to an incorrect target hypothesis, and delayed reaction, where the agent continues unnecessary exploration despite already having sufficiently strong target evidence.

The final action scoring weights $\lambda_{1},\lambda_{2},\lambda_{3},$ and $\lambda_{4}$ are determined through validation-based tuning. These coefficients balance four factors in the final decision: reasoning preference, prior-knowledge consistency, exploration utility, and estimated goal approach. The final parameter setting is chosen to achieve stable performance across Object-Goal, Image-Instance Goal, and Text-Goal navigation, rather than overfitting to a single goal modality.

For the knowledge-fusion temperature τ, we use a fixed value selected on the validation set to balance sharpness and stability in attention-based prior injection. A smaller temperature makes knowledge selection more concentrated but may increase sensitivity to noisy retrieval, whereas a larger temperature produces smoother aggregation but may weaken the effect of highly relevant priors. Therefore, τ is chosen to provide stable graph enhancement across different scene conditions.

### Appendix A.5. Qualitative Analysis of Representative Successful and Failure Cases

To provide deeper insights into the strengths and limitations of PriorNav, we present representative successful and failure navigation cases.

#### Appendix A.5.1. Successful Case

In the successful case, the target reference image describes a white toilet surrounded by typical bathroom objects, including a sink, mirror, towel rack, and tissue box. This target has strong semantic and relational constraints. Semantically, a toilet is highly associated with a bathroom; relationally, a toilet commonly co-occurs with bathroom facilities such as a sink, mirror, towel rack, and tissue box; at the instance level, the target has a white appearance, a curved contour, and a typical toilet structure. Therefore, this case requires not only recognizing the visual appearance of the target itself, but also using surrounding bathroom objects to verify the target region through contextual evidence.

As shown in Figure A1, the agent is not initially located near the target toilet. Instead, it first observes indoor structures such as walls, doors, a bedroom entrance, a bed, a sofa, a corridor, and adjacent rooms. At this stage, the target is not directly visible, and PriorNav mainly operates in the exploration stage. According to our method, the system constructs a task-aware query based on the current RGB-D observation, the target reference image, and the navigation history, and retrieves target-relevant semantic, instance-level, and relational priors from the unified knowledge embedding space. For the “toilet” target, semantic knowledge helps the agent infer that bathroom regions should be assigned higher search priority, whereas bedrooms, living rooms, and kitchens, although useful for completing the environmental map, are less semantically consistent with the target.

As navigation proceeds, the agent gradually moves from the bedroom and corridor areas to regions containing a red carpet, cabinets, a refrigerator, kitchen facilities, and a bathroom entrance. Figure A1 shows that the agent does not simply move along the shortest traversable path; instead, it progressively expands the explored area on the map and adjusts its search direction according to the observed functional regions. When objects such as a sink, mirror, cabinet, narrow doorway region, and bathroom facilities appear in the observation, they are added as nodes to the dynamic scene graph and connected to other indoor objects through spatial or semantic relation edges. Unlike methods that rely only on local matching in the current visual frame, PriorNav injects retrieved prior knowledge into the scene graph through knowledge attention, allowing the scene representation to preserve both online perceptual evidence and target-relevant prior context.

In this successful case, relational knowledge plays a particularly important role. The target reference image provides a clear bathroom-context cue involving objects such as a toilet, sink, mirror, towel rack, and tissue box. When the agent gradually observes the sink, mirror, and local bathroom area, the alignment between the knowledge-enhanced scene graph and the goal graph increases the target relevance of these regions, thereby raising the target confidence $\gamma_{t}$. In other words, the system is not merely searching for a white object, but verifying whether the white candidate object is located within a reasonable bathroom context. This helps exclude other white or light-colored distractors, such as walls, cabinets, refrigerators, or the sink itself, because although they may be similar in color to the target, they lack the structural form and relational context corresponding to a toilet.

In the verification stage, PriorNav further uses scene-goal alignment to filter candidate regions. Later frames in Figure A1 show that the agent gradually approaches the bathroom region, where the RGB observations begin to contain clearer structures around the sink, mirror, cabinet, and toilet. At this stage, instance-level knowledge is used to confirm the white appearance, toilet contour, and local geometric structure of the target, while relational knowledge is used to verify whether the spatial relations between the candidate object and surrounding objects such as the sink and mirror are consistent with the target image description. As these pieces of evidence become increasingly consistent, the system transitions from context verification to the approach stage and executes more precise target-approaching actions.

The agent successfully reaches the target toilet region, achieving SR = 1.000 and SPL = 0.422. This case shows that when the target has a clear semantic location prior and stable relational context, PriorNav can effectively exploit the complementary effects of the three types of prior knowledge: semantic knowledge narrows the search space and prioritizes bathroom regions; relational knowledge verifies candidate regions through contextual objects such as the sink, mirror, towel rack, and tissue box; and instance-level knowledge finally confirms the white toilet instance. By organizing these cues into a knowledge-enhanced scene graph and combining them with multi-step iterative reasoning for stage-adaptive decision-making, PriorNav can achieve stable and efficient instance-level target navigation in unseen environments.

#### Appendix A.5.2. Failure Case

In the failure case, the target reference image describes a wooden-and-black chair. The target reference image shows no obvious surrounding objects or decorations around the chair, suggesting that it may be located in a kitchen, dining area, or another region with relatively sparse surrounding furniture. Compared with the white toilet in the successful case, this target has weaker relational constraints and greater location uncertainty. A chair may appear in a dining room, kitchen, living room, bedroom, near a desk, or in a transitional corridor area, while the absence of obvious surrounding objects does not provide a stable co-occurrence relation. Therefore, this case is more susceptible to scene ambiguity, appearance-level distractors, and fluctuations in target confidence.

As shown in Figure A2, the agent first observes a bathroom region in the early navigation stage, including a sink, toilet, shower curtain, door, and walls. For the “wood-and-black chair” target, these bathroom objects have weak semantic relevance to the goal, and thus semantic knowledge should guide the agent to continue searching for regions where a chair is more likely to appear. Subsequently, the agent passes through corridors and room entrances and gradually enters the bedroom, kitchen, dining area, and living room. Figure A2 shows many wooden doors, wooden cabinets, dining tables and chairs, kitchen facilities, curtains, living-room furniture, and dark- or wood-colored objects. These objects may all be related to the target chair to some extent in terms of semantics, color, or material, thereby forming multiple candidate search regions.

The first major difficulty in this failure case is that semantic knowledge does not provide a sufficiently unique constraint. For the toilet target, semantic knowledge can relatively clearly narrow the search space to the bathroom. In contrast, chairs have more dispersed possible locations, including the dining room, kitchen, living room, bedroom, or desk area. Therefore, when the agent observes regions such as the kitchen, dining area, living room, and bedroom, all of them may be considered partially relevant to the target. Although this prior knowledge can prevent the agent from remaining in obviously irrelevant regions such as the bathroom for too long, it cannot eliminate most candidate spaces as effectively as in the toilet case, leading to a larger search range and lower path efficiency.

This case is more strongly affected by interference in instance-level matching. The key visual cues of the target chair are its wooden texture and black components, while the scene contains many visually similar distractors, such as wooden doors, wooden cabinets, dining tables, dining chairs, kitchen cabinets, dark furniture edges, and dark objects in the living room. These objects may be similar to the target image in local color, material, or edge structure, but they are not necessarily the target chair instance. For Image-Instance Goal Navigation, the agent must identify the specific instance in the reference image rather than any chair. Therefore, when multiple candidate objects simultaneously exhibit wooden textures, black local structures, or chair-like shapes, the discriminative difficulty of instance-level knowledge increases substantially.

Relational knowledge in this case provides a weaker verification signal. The toilet target in the successful case has explicit co-occurrence relations, such as the sink, mirror, towel rack, and tissue box, which can stably support target verification through scene-graph relation edges. In contrast, the chair target in the failure case is described as having no obvious surrounding objects or decorations, which is essentially a weak or negative relational constraint. It requires the system to determine whether there are few salient contextual objects near the candidate chair, rather than verifying whether the chair stably co-occurs with certain specific objects. For a knowledge-enhanced scene graph, explicit co-occurrence relations are easier to model and use, whereas constraints such as a locally sparse environment or few surrounding objects are more vulnerable to viewpoint changes, occlusion, and incomplete map coverage. Therefore, even though PriorNav can construct a dynamic scene graph, it may still be difficult to clearly distinguish the true target chair from other wooden or black furniture using relational priors alone.

In addition, the later trajectory in Figure A2 shows that the agent conducts substantial exploration in the kitchen, dining area, and living room, and approaches several furniture regions that may be related to the target. This indicates that the system can use prior knowledge to shift the search from the bathroom to more plausible indoor functional areas, but it remains difficult to form a stable target hypothesis among multiple candidate regions. According to the stage-adaptive reasoning mechanism in our method, the target confidence $\gamma_{t}$ determines the transition among the exploration, verification, and approach stages. When multiple candidate objects share a certain degree of semantic or visual similarity with the target, $\gamma_{t}$ may fluctuate around the stage-transition thresholds, causing the agent to repeatedly trade off continued exploration, candidate verification, and target approach. If an incorrect candidate region receives a high goal-approach score, the agent may prematurely move toward a non-target region; if the exploration utility remains dominant, the agent may produce a longer detour and repeated search behavior.

This case obtains SR = 0.667 and SPL = 0.302, indicating that the agent may have approached a target-related region but failed to fully satisfy the success condition, or that there was a deviation in final target confirmation and the stop action. This failure does not indicate that PriorNav is unable to handle chair targets; rather, it reveals a more challenging situation in Image-Instance Goal Navigation: the target category has dispersed possible locations, the target appearance is similar to multiple objects in the scene, and the target description lacks a stable positive relational context. In such cases, it is difficult for semantic knowledge, instance-level knowledge, and relational knowledge to form a strongly consistent target hypothesis, which further affects scene-goal alignment, target-confidence estimation, and final action scoring.

Overall, the successful and failure cases jointly demonstrate the advantages and boundary conditions of PriorNav. In the successful case, the toilet target has a clear semantic location prior and stable bathroom co-occurrence relations, allowing PriorNav to rapidly reduce the search space through semantic knowledge, verify the target context through relational knowledge, and complete final instance confirmation through instance-level knowledge. In the failure case, the chair target has more dispersed possible locations, its wood-and-black appearance is easily confused with wooden doors, cabinets, dining tables and chairs, and dark furniture, and the constraint of having few surrounding objects is less stable than explicit co-occurrence relations, making target-confidence estimation and action selection more difficult. These qualitative results complement the quantitative experiments and ablation studies in the main paper, further showing that the performance gains of PriorNav arise from the synergy among prior knowledge, structured scene representation, and stage-adaptive reasoning, while also revealing key aspects that require further improvement in complex real-world scenes.
